# The role of FOXA1 and miR-212-3p in molecular modulation of doxorubicin resistance in liver cancer

**DOI:** 10.1007/s12032-025-02686-5

**Published:** 2025-04-11

**Authors:** Ammar Elfiky, Nadia El-Guendy, Abeer Mahmoud Badr, Mohammed Aly Mohammed, Abdel Hady A. Abdel Wahab

**Affiliations:** 1 Medical Biochemistry and Molecular Biology at Cancer Biology Department National Cancer Institute, Cairo University, Giza, Egypt; 2 Zoology Department, Faculty of Science, Cairo University, Giza, 12613 Egypt

**Keywords:** Liver cancer, FOXA1, Drug resistance, MicroRNA, Doxorubicin

## Abstract

TACE (Transarterial Chemoembolization) is an essential current treatment for liver cancer. Resistance to doxorubicin, the chemotherapeutic component of TACE, poses a serious problem in this treatment, necessitating a deeper understanding of the underlying resistance mechanisms. Upregulation of the Forkhead box A1 transcription regulator in our model of doxorubicin-resistant liver cancer cell line suggested a role in resistance. To better understand the role of FOXA1 in resistance to doxorubicin, we inhibited its expression using siRNA or its miRNA-212-3p inhibitor then studied the effect on the cancer cell lines survival using SRB assay. The expression of several downstream epithelial-mesenchymal transition genes, namely *SLUG*, *TWIST*, *CDH1* (E-Cadherin), was determined using quantitative real-time PCR. Our results showed a significant upregulation of FOXA1 and downregulation of miRNA-212-3p in doxorubicin-resistant cells. Manipulation of FOXA1 and miRNA-212-3p expressions restored sensitive cell characteristics. In addition, inhibition of FOXA1 increased apoptosis induction in resistant cells. Changes detected in the tested EMT genes point to progression toward more aggressive behavior in the doxorubicin-resistant liver cancer cell line that was reversed with inhibition of FOXA1. Our results suggest a possible role of FOXA1 and miRNA-212-3p in the development of resistance to chemotherapeutic drugs in liver cancer and the possibility of their use as prognostic and/or therapeutic targets.

## Introduction

Liver cancer is the sixth most common cancer globally and the fourth leading cause of cancer-related deaths, with approximately 841,000 new cases and 782,000 deaths annually. It is a major health concern in Egypt’s cancer profile. As per the Global Cancer Observatory’s 2022 data, it is the second most prevalent cancer in the country, following breast cancer. With an age-standardized incidence rate (ASR) of 32.0 per 100,000 individuals, liver cancer in Egypt ranks among the highest worldwide. While global treatment protocols have evolved to favor systemic therapies, such as tyrosine kinase inhibitors (TKIs) and immunotherapies for advanced liver cancer, Egypt continues to rely heavily on transarterial chemoembolization (TACE) treatment modality. This approach is primarily due to the limited availability and high costs associated with newer systemic treatments. TACE, often utilizing doxorubicin, has demonstrated efficacy in managing intermediate-stage liver cancer within the Egyptian healthcare context [1–3]. Doxorubicin, a widely used anticancer therapy that interferes with topoisomerase IIα (TOP2A) function, inducing the stabilization of cleaved-strand intermediates and hampering DNA strand separation, thereby generating protein-bound double-strand breaks (DSBs) [4]. Despite the proven efficacy of doxorubicin in liver cancer treatment, the latter remains notably resistant to chemotherapy. Thus, chemotherapy resistance stands as a major challenge, significantly limiting the successful treatment of patients with liver cancer [5].

A notable breakthrough in the treatment of hepatocellular carcinoma (HCC), the most prevalent form of primary liver cancer, involves the targeted delivery of cytotoxic chemotherapy agents directly to the tumor via TACE [6]. This approach, particularly when using doxorubicin, enhances the local concentration of the drug while minimizing systemic exposure, leading to improved therapeutic outcomes. While tumor embolization alone induces ischemia and tumor shrinkage, doxorubicin-based TACE has played a crucial role in downsizing tumors for liver transplantation eligibility, contributing significantly to therapeutic advancements [7]. However, resistance to doxorubicin poses a significant challenge, limiting the efficacy of TACE. Despite its success in many patients, approximately 50% of tumors treated with doxorubicin-eluting beads (DEB-DOX) show no response, with only 27% achieving a complete response. Understanding the mechanisms behind doxorubicin resistance is essential for enhancing treatment efficacy and developing novel or supplementary strategies to improve outcomes in liver cancer patients [8].

Resistance to chemotherapy in cancer cells can arise through various mechanisms [9], including altered membrane transport through transporters like ABCB1 (MDR1) [4], enhanced DNA repair [10], defects in apoptotic pathways [11], and alterations in target molecules, proteins, and cell cycle regulation pathways [12]. Epithelial–mesenchymal transition (EMT) is recognized as a significant contributor to drug resistance [13]. MicroRNAs (miRNAs) have gained recent attention as potential markers of chemotherapy resistance [14].

Our previous studies on doxorubicin-resistant liver cancer cell lines identified the Fork head box A1 (*FOXA1*) gene as a possible player in doxorubicin resistance. FOXA1, a transcriptional activator within the fork head box family, regulates key genes involved in tumor growth, differentiation, apoptosis, metastasis, invasion, and drug resistance. While the relationship between *FOXA1* and EMT genes has been explored in prostate and breast cancers, its role in liver cancer resistance has not been deeply studied before. Bioinformatic analysis identified miRNA-212-3p as a regulator of FOXA1, prompting further investigation. [4, 15, 21].

Our study aims to assess the impact of doxorubicin on the survival of liver cancer cell lines after knocking down FOXA1 using an siRNA silencer or increasing inhibitory miRNA-212-3p expression.

Additionally, the study seeks to examine the downstream EMT genes: *CDH1* (E- cadherin), *SLUG*, and *TWIST*, as potential targets of FOXA1. These genes are crucial regulators of the EMT process in promoting cancer invasion and metastasis. E-CADH (E-cadherin) plays a vital role in maintaining cell–cell adhesion in epithelial tissues, and its downregulation is associated with increased invasiveness in cancer cells. SLUG and TWIST are key transcription factors that repress E-CADH expression, driving EMT and contributing to tumor progression. Their expression is frequently altered in liver cancers, which makes them critical markers of EMT and potential indicators of therapeutic resistance.

We also investigated the effect of modulating FOXA1 and miRNA-212-3p on P53 and BCL2 expression levels. P53 plays an essential in regulating cell apoptosis and tumor suppression, while BCL2 is involved in inhibiting cell death and promoting drug resistance [4, 15].

Investigating the expression changes in these EMT markers and resistance-related genes will help understand the pathways involved in doxorubicin resistance and potential therapeutic strategies.

## Materials and methods

### Cell line and reagents

The human liver cancer cell line, HepG2 was obtained from VACSERA (the Egyptian Company for Production of Vaccines, Sera and Drugs). HepG2 cell line was grown as a monolayer culture in RPMI-1640 high glucose- medium (Sigma Aldrich, USA), supplemented with 10% fetal Bovine Serum (FBS), 100 U/ml penicillin, and 100 mg/ml streptomycin (Gibco, USA).

The RNA purification kit (miRNeasy Mini Kit), the miScript RNA reverse transcription kit (miScript II RT Kit), and the miScript SYBR Green PCR kit were all obtained from Qiagen (Qiagen, Germany). All primers were sourced from Qiagen, Germany.

FOXA1 Silencer® siRNA, miR-212-3p Ambion® Pre-miR™ miRNA Precursor, and their respective Ambion™ Negative Controls siRNA were purchased from Thermo Fisher Scientific.

Sub-culturing was performed when cells reached 90–95% confluency. Sub-culturing was performed when cells reached 90–95% confluency. The frequency of sub-culturing depended on the cells’ doubling time and their proliferation index.

### Cell viability determination

The potential cytotoxicity of doxorubicin on HepG2 cell line was determined using Sulforhodamine B (SRB) assay [16].

HepG2 and/or HepG2/Dox cells were seeded in 96-well plate at a concentration of 3 × 10 [3] cells/well in fresh medium. After 24 h. cells were incubated with different doxorubicin concentrations (0.5, 2, 4, 5, and 8 μg/ml) for 48 h.

SRB assay was performed as previously described to determine the cell viability and to calculate the IC50 values and fold resistance. Briefly, treated cells, which were fixed with trichloroacetic acid (TCA) and stained with SRB dye that binds to basic amino acids of cellular proteins. After removing the unbound dye, the retained SRB was quantified to assess cell viability in response to different doxorubicin concentrations. This data was used to determine cell viability, calculate IC50 values, and determine the fold resistance.

### Development of doxorubicin-resistant HepG2 cells (HepG2/Dox)

To mimic the situation of HCC cells surviving TACE treatment, a doxorubicin-resistant HepG2 cell line was established using single step doxorubicin treatment as described previously [17]. Briefly, HepG2-resistant cells were selected by treating HepG2 cells with an IC80 dose (16 µg/ml ~ 29.4 µM) of doxorubicin, which kills 80% of the cells over 48 h. Subsequently, the medium was replaced with fresh, complete doxorubicin-free medium. The remaining 20% of surviving cells were resistant to doxorubicin treatment. Surviving HepG2 cells (HepG2/Dox) and parental HepG2 cells were further cultured in complete doxorubicin-free medium, with regular passage for 3 more weeks before proceeding with subsequent experiments.

### Transfection

To evaluate the effect of FOXA1 on cell’s resistance to doxorubicin, we overexpressed miRNA-212-3p, or inhibited FOXA1 expression in HepG2/Dox cells by transfecting with miRNA-212-3p mimic, FOXA1 silencer, or negative controls with or without doxorubicin treatment using HiPerFect transfection Kit.

HepG2/Dox Cells were seeded in a 6-well plate (3 × 10^5^ cells per well) and incubated under normal growth conditions (typically 37 °C and 5% CO2). Prior to transfection, the medium was replaced with 1600 μl complete media.

The following transfection complexes were prepared in 400 μl serum free media: FOXA1 silencer and NCf (Negative control of FOXA1 silencer) at a final concentration of 50 nm with HiPerFect Reagent (3 μl), miRNA212-3p MIMIC and NCm (Negative control of mimic) at a final concentration of 10 nm with HiPerFect Reagent (3 μl). The cells were incubated with the transfection complexes of (mimic or silencer) with their respective negative controls in triplicates, alone or in combination with IC50 dose of Doxorubicin. The transfected/ treated and untransfected cells were harvested for analyses after 48 h.

### Quantitative real-time polymerase chain reaction assay (qPCR)

qPCR was performed to determine the expression levels of target genes in HepG2, and HepG2/Dox cells. Total RNA extraction was performed using miRNeasy Mini Kit from harvested cells. The quantity of the extracted RNA was measured by Nano Drop (Thermo-Fisher, USA).

Total RNA was reverse transcribed to cDNA using miScript II RT Kit. The qPCR was performed in ViiA7 real-time PCR system from Applied Biosystems, USA, using miScript SYBR Green PCR kit. The cycling conditions for the mRNA amplification were 95 °C 10' (95 °C 15", 63 °C 30", 70 °C 30") X45, and for miRNA 212-3P 95 °C 10' (95 °C 15", 55 °C 30" , 70 °C 30") X45. The 2^−ΔΔCT^ method was used as a relative quantification strategy for qPCR data analysis [18]. The mRNA data was normalized to GABDH while the miRNA ones were normalized to RNU6B. All primers used are in Table 1**.**

**Table 1:** Primers of genes of interest

Gene	Forward	Reverse
*GAPDH*	5'CCATGGAGAAGGCTGGGG3'	5'CAAAGTTGTCATGGATGACC3'
*RNU6B*	5'GCCCCTGCGCAAGGATGAC3'	Universal stem-loop primer: 5′GAAAGAAGGCGAGGAGCAGATCGAGGAAGAAGACGGAAGAATGTGCGTCTCGCCTTCTTTCNNNNNNNN-3′
*FOXA1*	5'CCATGGAGAAGGCTGGGG3'	5'CAAAGTTGTCATGGATGACC3'
*miRNA-212-3p*	5'CAGTCTCCAGTCACGG3'	Universal stem-loop primer: 5′GAAAGAAGGCGAGGAGCAGATCGAGGAAGAAGACGGAAGAATGTGCGTCTCGCCTTCTTTCNNNNNNNN-3′
*MDR (ABCB1)*	5'GCTGTCAAGGAAGCCAATGCCT3'	5'TGCAATGGCGATCCTCTGCTTC 3'
*CDH1*	5'GGTTTTCTACAGCATCACCG3'	5'GCTTCCCCATTTGATGACAC3'
*SLUG*	5'TGTCAAAAGTGTGAGAGAAT3'	5'CTTGCCAGCGGGTCTGGC3'
*TWIST*	5'CGCGGGGGACGCAGGCGGCGCAGCAGC3'	5'GCTGCTGCGCCGCCTGCGTCCCCCGCG3'
*TP53*	5'CACAGCACATGACGGAGGTC3'	5'TCCTTCCACCCGGATAAGATG3'
*BCL2*	5′TCCGATCAGGAAGGCTAGAGTT-3′	5′TCGGTCTCCTAAAAGCAGGC-3′

### Apoptosis analysis using flow cytometry

Cell apoptosis was determined using an Annexin-V fluorescein isothiocyanate (FITC) apoptosis detection kit (Biospes, Cat# BAD1001). HepG2/Dox cells were incubated with the transfection complexes of (miRNA-212-3p mimic or FOXA1 silencer or their negative control in triplicates for 48 h, alone or in combination with IC50 dose of Doxorubicin. The transfected/treated cells were harvested for flow cytometric measuring, and as per the kit’s instructions, they were centrifuged for 10 min at 1800 rpm, then resuspended in PBS. The mixture was centrifuged again for 10 min at 1200 rpm and the cell suspension was adjusted to a concentration of 1X 10^6^ cells per ml. Cells were stained with 5 μL of Annexin-V for 15 min at 4 °C in the dark. Analysis of 10,000 events was performed using flow cytometry Beckman Coulter, Navios, equipped with 525/40 filter detector (FL1) for apoptosis detection. Data analysis was done using kaluza analysis version 2.1 software.

### Statistical analysis

Data are presented as mean ± standard deviation (SD). Multiple comparisons were carried out using one-way analysis of variance (ANOVA) followed by Tukey test. Graphs were performed using Prism software 5, CA, USA, and analysis of data was performed using GraphPad INSTAT (version 3). The two-tailed Student’s t-test was used for testing a hypothesis since a difference between two groups. Statistical significance was acceptable at a level of P-value = < 0.05.

## Results

### Development of doxorubicin-resistant HepG2 cells (HepG2/Dox)

**Fig. 1:**
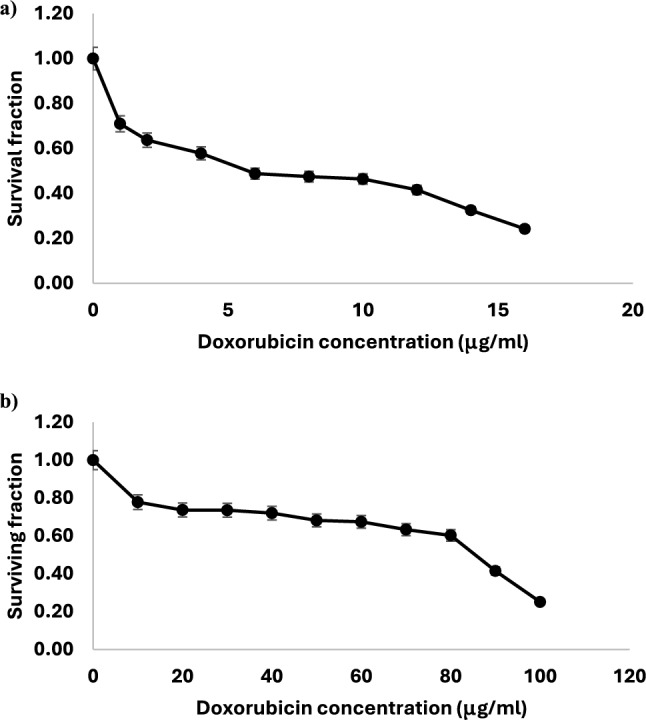
Doxorubicin IC50 dose determination **a** Parental HepG2 cells. **b** HepG2/ Dox cells. The graph shows the surviving fraction of cells treated with different concentrations of doxorubicin (0–16 μg/ml) and (0–100 μg/ml) for 48 h. The results are expressed as the mean ± SD of 3 separate experiments

The 50% inhibition concentration value (IC50) of doxorubicin and the IC80 were determined to be 5.4 μg/ml (9.9 µM) and 16 µg/ml (29.4 µM), respectively, using the SRB technique, **(**Fig. 1a).

Doxorubicin-resistant HepG2 cells (HepG2/Dox) were developed by subjecting the cells to a single high dose (IC80) of doxorubicin as described previously [17].

### Characterization of HepG2/Dox

#### HepG2/Dox cells are 16-fold more resistant to doxorubicin

To confirm the resistance characteristics of the newly developed cell line, the resistance index (RI), which is the IC50 for HepG2/Dox divided by the IC50 of HepG2 (HepG2/Dox/IC50), was determined using the SRB assay. This experiment revealed that the HepG2/Dox cells were about 16-fold more resistant to killing by doxorubicin than parental cells. Figure 1b shows the effect of different concentrations of doxorubicin on the cellular proliferation of resistant HepG2/Dox cell line after 48 h of treatment. As shown, doxorubicin produced a dose dependent decrease in cell viability compared to the non-treated control. The 50% inhibition concentration value (IC50) of doxorubicin for the resistant HepG2/Dox cell line was 86 μg/ml as opposed to 5.4 μg/ml for parental HepG2.

#### Decrease in apoptosis in HepG2/Dox cells

**Fig. 2:**
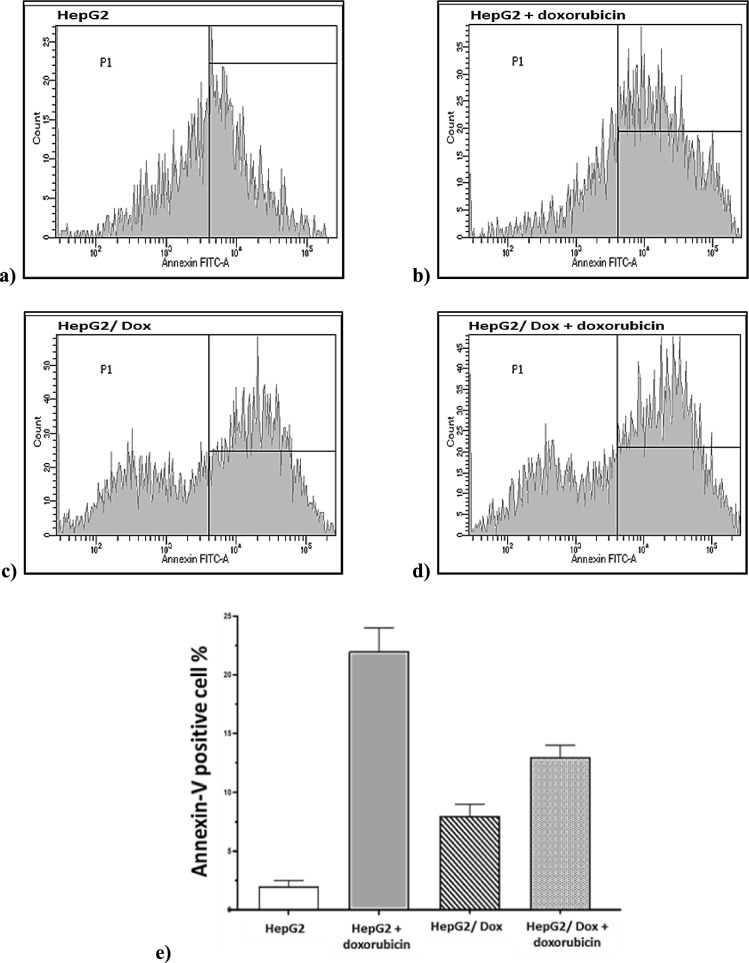
Apoptosis of HepG2 and HepG2/Dox in response to doxorubicin treatment cells. Panels **a** to **d** show Annexin-V positive cell percentage in HepG2 and HepG2/Dox cells untreated or treated with IC50 dose of doxorubicin for 48 h using flow cytometry. **e** shows a graph comparing Annexin-V expression percentage between the cell groups

To confirm the resistance characteristics of HepG2/Dox cells, we measured the percentage of apoptosis in response to the parental cells’ IC50 doxorubicin dose of 5.4 μg/ml. Flow cytometry analysis of Annexin-V-treated cells revealed that apoptosis induction was four times lower in resistant HepG2/Dox cells (13% of treated resistant cells were Annexin-V positive vs. 8% in control resistant cells) compared to parental HepG2 cells (22% of treated parental cells were Annexin-V positive vs. 8% in control parental cells) at the IC50 dose. (Fig. 2).

#### Doxorubicin-resistant cells express high levels of FOXA1 and low levels of its inhibitor miRNA-212-3p

Previous unpublished transcriptomic data from our lab showed an increase in FOXA1 expression in HepG2/Dox cells. To confirm these results, we determined FOXA1 expression levels and its known miRNA-212-3p inhibitor in HepG2/Dox cells using qPCR.

**Fig. 3:**
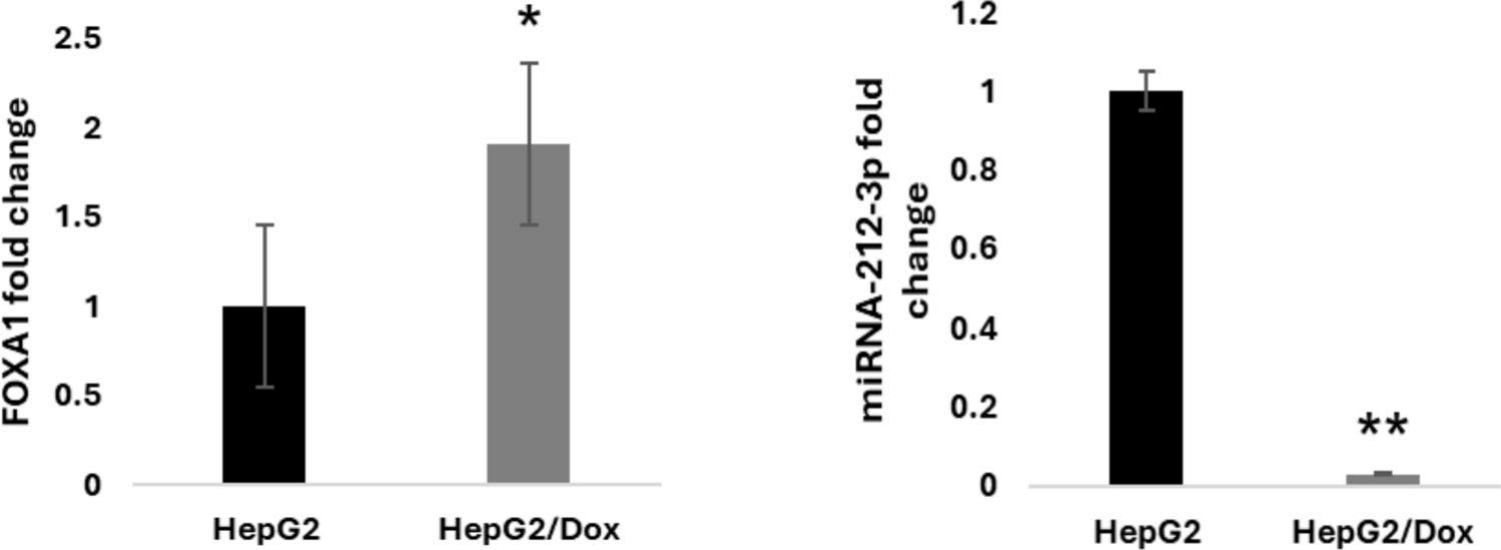
FOXA1 gene and its regulating miRNA-212-3p gene expression in HepG2/Dox: The expression levels were determined using qPCR assay for target genes. The data were normalized using GAPDH and RNU6B as endogenous controls, with parental HepG2 cells as the base control. Results are presented as fold change ± SD. The assay was performed in triplicate

The expression level of *FOXA1* gene was upregulated about twofold in HepG2/Dox compared to control parental HepG2 cells confirming our previous data (Fig. 3, Table 2). In addition, the expression level of its regulating miRNA (miRNA-212-3p) was downregulated 33-fold in HepG2/Dox compared to parental HepG2 cells.

**Table 2:** The expression levels of target genes in HepG2/Dox versus parental HepG2

Gene	Fold change for HepG2/Dox versus parental HepG2	t-test
*FOXA1*	1.91	0.0497*
*miRNA-212-3p*	0.03	0.0244**
*ABCB1*	9.9	0.0443*
*CDH1*	5.32	0.0022***
*SLUG*	33.4	0.0083**
*TWIST*	0.02	0.0135**
*TP53*	4.20	0.0492*
*BCL2*	2.03	0.1997

Data are represented as mean values ± SD from three replicates, significantly different (p-value = < 0.05). The statistical significance of the results was analyzed using the two-tailed Student’s t-test

#### Upregulation of ABCB1 expression level in HepG2/Dox

**Fig. 4:**
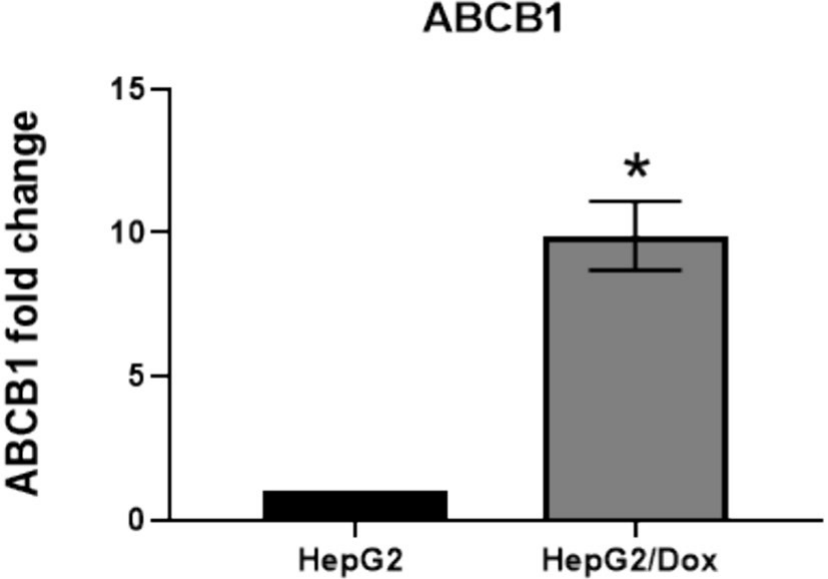
Expression level of ABCB1 in HepG2/ Dox: The expression level was determined using qPCR assay for the target gene. The data were normalized using GAPDH as the endogenous control, with parental HepG2 cells as the base control. Results are presented as fold change ± SD. The assay was performed in triplicate

We have previously shown that resistance of cancer cells to doxorubicin is associated with high level of the multidrug resistance protein 1 (MDR1/ABCB1) expression, which is due to its role in increasing the efflux of chemotherapeutic drugs. This protein plays a major role in drug resistance and is a major cause of treatment failure by extruding anticancer drugs as anthracyclines (doxorubicin) out of the cells [19, 20].To confirm the resistance characters of our cells, we measured the expression level of ABCB1 in resistant cell line HepG2/Dox versus parental HepG2 cell line using qPCR real-time assay. As expected, ABCB1 mRNA showed a significant upregulation in its expression level with a mean fold change of 10 in HepG2/Dox compared to control parental HepG2 cells (Fig. 4, Table 2).

#### Expression of some EMT genes is changed in HepG2/Dox

As FOXA1 plays a vital role in EMT progression in many cancers, such as breast and liver [21], we decided to investigate the expression levels of some FOXA1 downstream EMT genes. In addition to E-cadherin gene (*CDH1*), we selected 2 other EMT genes (*SLUG* and *TWIST*) which are all downstream of FOXA1.

**Fig. 5:**
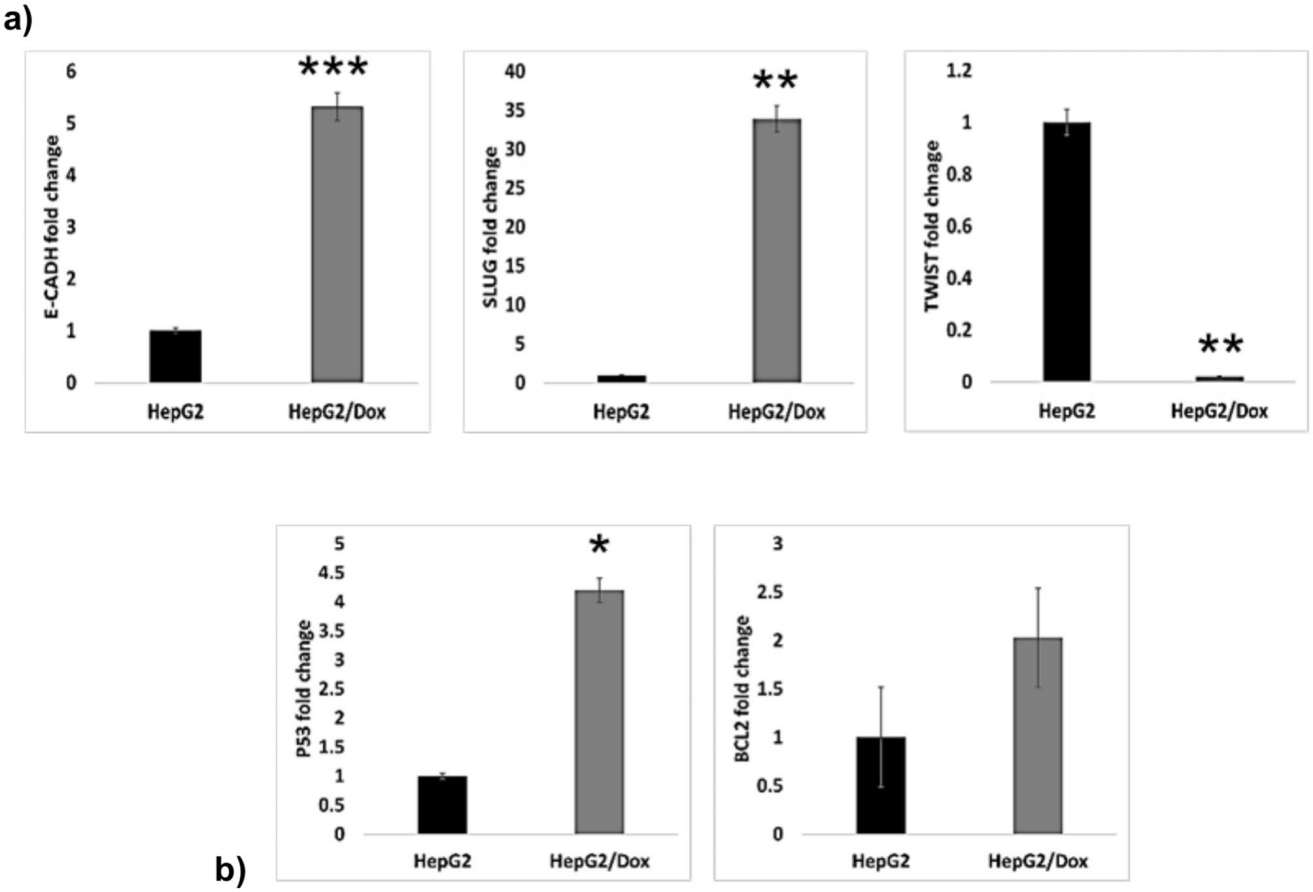
Gene expression in HepG2/Dox: The expression levels were determined using qPCR assay for target genes. **a** EMT genes *CDH1*, *SLUG,* and *TWIST*. **b** Apoptotic genes *TP53* and *BCL2*. The data were normalized using GAPDH as the endogenous control, with parental HepG2 cells as the base control. Results are presented as fold change ± SD. The assay was performed in triplicate

It was interesting to see if the changes caused by resistance of HepG2 to doxorubicin that led to increase in FOXA1 mRNA expression, also affected these EMT genes expression. We found that the expression levels of E-CADH and SLUG mRNA were upregulated fivefold and 33-fold, respectively, while the expression levels of TWIST were downregulated tenfold in HepG2/Dox compared to parental HepG2 cells (Fig. 5a, Table 2).

Increase in *SLUG* gene expression and downregulation of *CDH1* expression is usually associated with EMT progression [22], so the increase of SLUG mRNA seen in our resistant cells is consistent with the EMT progression and increase in drug resistance development. On the other hand, changes in E-cadherin (*CDH1)* are not consistent with the known changes in the EMT process, since progression of cancer development is accompanied by cell–cell contact inhibition and high invasiveness, resulting from reduced intercellular adhesion [23]. However, this discrepancy has been described in Wei et al. study [24] were they found that about 30% of HCC samples show downregulation of E-cadherin while 40% shows upregulation and that this elevation positively correlated with invasive potential of tumor cells.

#### Changes in the expression levels of *TP53* and *BCL2* genes in HepG2/Dox

The expression level of *TP53* and *BCL2* genes was upregulated fourfold and twofold, respectively, compared to parental HepG2 cells (Fig. 5b, Table 2).

The upregulation of the apoptotic gene *TP53*, despite the decrease of apoptosis in resistant cells, could be linked to the formation of double-strand DNA breaks (DSBs) because of doxorubicin exposure [25]. Since P53 is known to cause cell cycle arrest, its upregulation in our study could be the result of the exposure to a lethal dose of doxorubicin and increase of DSB. The upregulated modulator of apoptosis BCL2 is involved in the mitochondrial apoptosis pathway activated by drugs, such as sorafenib and cabozantinib in HCC [26]. The upregulation of BCL2 mRNA in our study suggests that changes causing resistance to doxorubicin may affect the mitochondrial apoptotic pathway in liver cancer cell lines.

#### Inhibition of FOXA1 increases sensitivity to Doxorubicin-induced apoptosis in HepG2/Dox cells

**Fig. 6:**
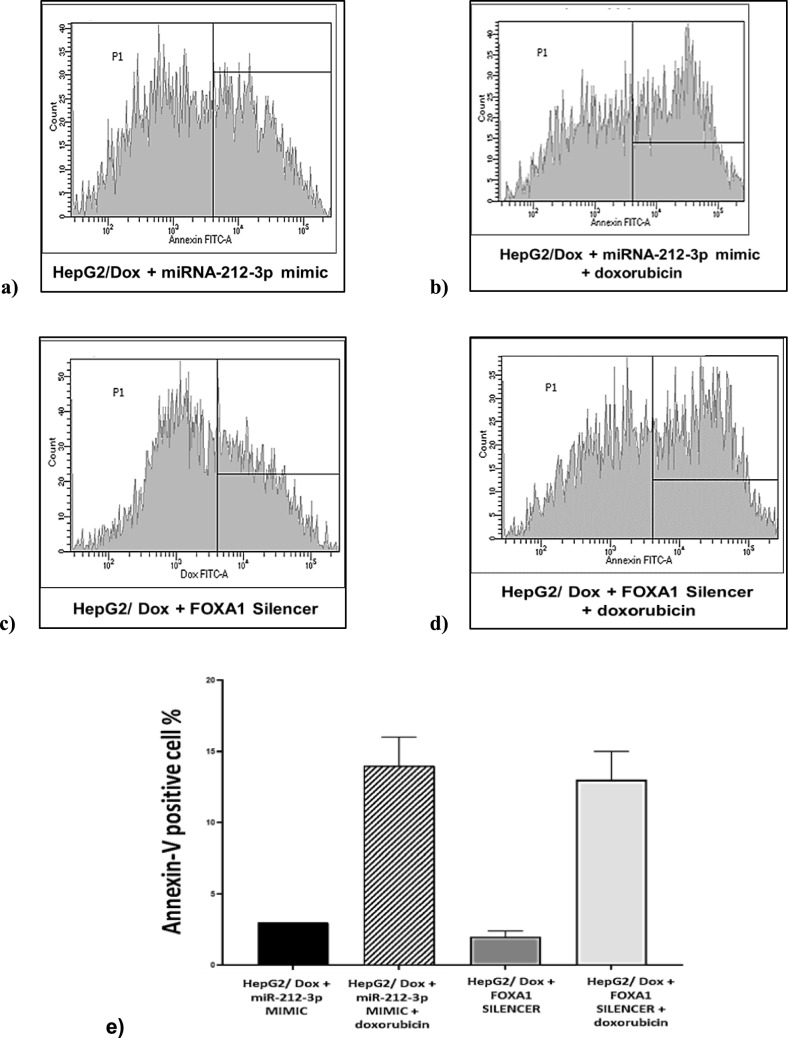
Flow Cytometric detection of apoptosis in HepG2/Dox transfected cells. **a** Percentage of Annexin-V positive cells in HepG2/Dox cells transfected with miRNA-212-3p mimic. **b** Percentage of Annexin-V positive cells in HepG2/Dox cells transfected with miRNA-212-3p mimic and treated with IC50 dose of doxorubicin. **c** Percentage of Annexin-V positive cells in HepG2/ Dox cells transfected with FOXA1 silencer. **d** Percentage of Annexin-V positive cells in HepG2/Dox cells transfected with FOXA1 silencer and treated with IC50 dose of doxorubicin. Graphs **a-d** represent the mean fluorescent intensity of Annexin-V. Graph** e** shows the Percentage of Annexin-V positive cells (apoptotic cells) in transfected HepG2/Dox cells groups after doxorubicin treatment for 48 h using flow cytometry

To further assess the effect of FOXA1 inhibition on the sensitivity of HepG2/Dox cells to Doxorubicin, apoptosis induction was determined in transfected cells treated with Dox for 48 h. Apoptosis was assessed by measuring Annexin-V binding using flow cytometry [27] (Fig. 6).

Apoptosis induction increased up to fourfold in both the miRNA-212-3p MIMIC + IC50 doxorubicin group (14% of transfected treated cells were Annexin-V positive vs. 3% in control cells) and the FOXA1 silencer + IC50 doxorubicin group (13% of transfected treated cells were Annexin-V positive vs. 2% in transfected control cells). Downregulating the FOXA1 via siRNA silencer transfection or increasing its inhibition through miRNA-212-3p expression significantly enhanced apoptosis induction in resistant HepG2/Dox cells in response to the IC50 dose (5.4 μg/ml) of doxorubicin. These findings suggest a potential role of FOXA1 and miRNA-212-3p in chemotherapy resistance in HepG2/Dox cells and their involvement in restoring drug sensitivity.

#### Inhibition of FOXA1 affects the expression of ABCB1, EMT, and Apoptosis-related genes

To determine the effect of FOXA1 on doxorubicin resistance, we used FOXA1 inhibitor miRNA-212-3p and FOXA1 silencer to modulate its expression.

**Fig. 7:**
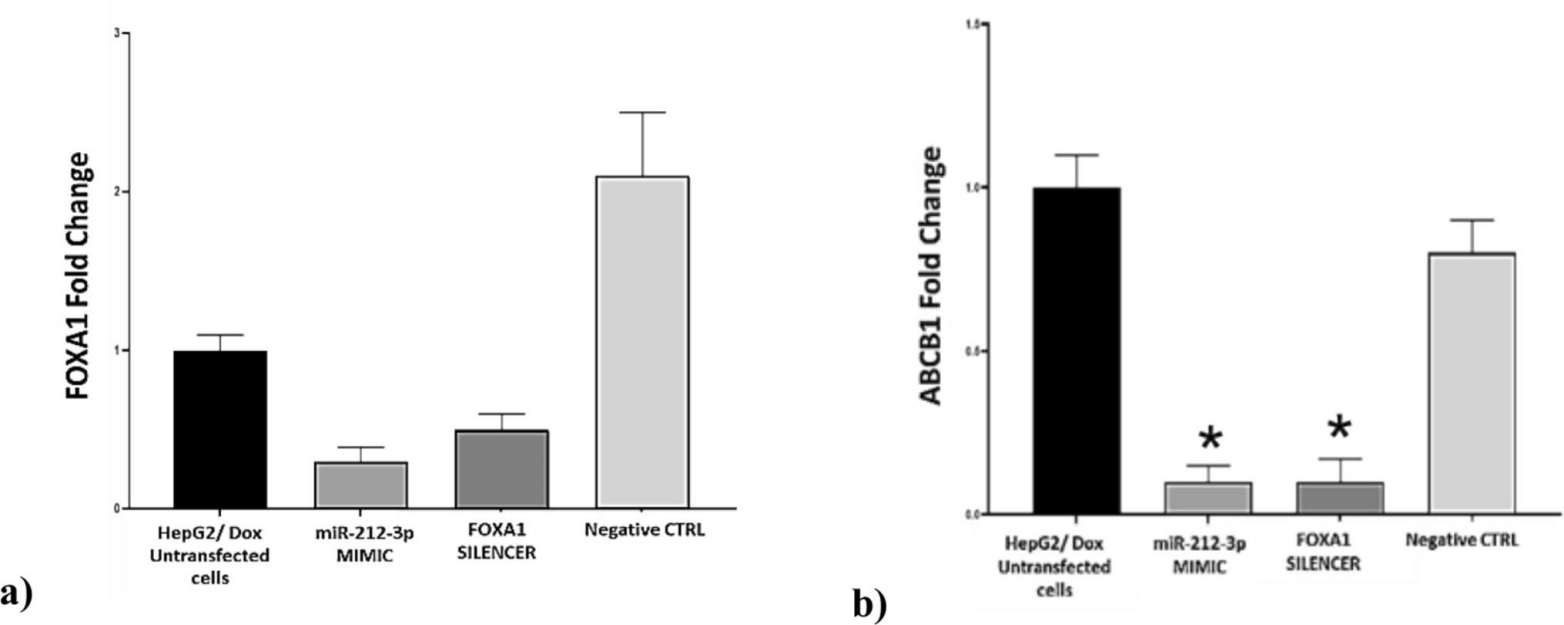
qPCR assay for the target gene in transfected HepG2/ Dox **a** Expression level of *FOXA1*. **b** Expression level of *ABCB1*. The expression level was determined using qPCR assay for the target gene. The data were normalized using GAPDH as the endogenous control, with HepG2/ Dox cells as the base control. Results are presented as fold change ± SD. The assay was performed in triplicate

**Fig. 8:**
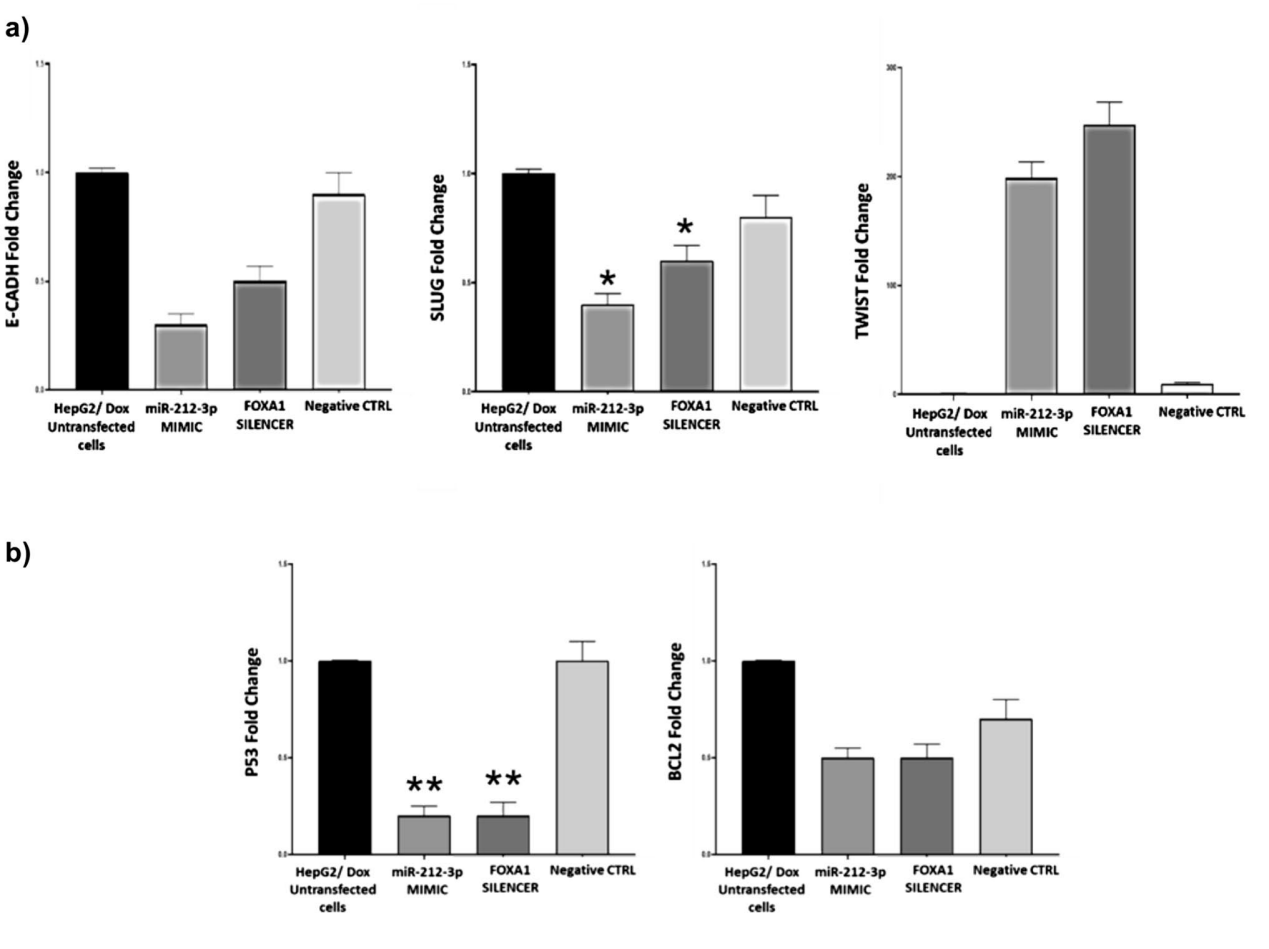
Gene expression in transfected HepG2/Dox: The expression levels were determined using qPCR assay for target genes. **a** EMT genes *CDH1*, *SLUG,* and *TWIST*. **b** Apoptotic genes *TP53* and *BCL2*. The data were normalized using GAPDH as the endogenous control, with untransfected HepG2/Dox cells as the base control. Results are presented as fold change ± SD. The assay was performed in triplicate and repeated at least three times

FOXA1 expression was downregulated fourfold in miRNA-212-3p MIMIC transfected cells, and up to twofold in FOXA1 silencer transfected cells compared to the untransfected control (Fig. 7a). Noting that the expression level of FOXA1 gene was significantly upregulated twofold with the development of resistance to doxorubicin as shown in (Fig. 4, Table 2). Interestingly, ABCB1 expression was downregulated eightfold in miRNA-212-3p MIMIC group, and 14-fold in FOXA1 silencer group compared to the untransfected control (Fig. 7b). Noting that ABCB1 expression was found to be elevated up to tenfold with increased resistance to doxorubicin as shown in (Fig. 4, Table 2). As for the EMT-related genes, *CDH1* expression was downregulated threefold with miRNA-212-3p MIMIC, and twofold with FOXA1 silencer compared to the untransfected control (Fig. 8a). Noting that *CDH1* expression was found to be elevated up to fivefold with increased resistance to doxorubicin as shown in (Fig. 5a, Table 2). SLUG mRNA expression was downregulated threefold in miRNA-212-3p MIMIC group, and twofold in FOXA1 silencer group compared to the untransfected control (Fig. 8a). Noting that SLUG expression was found to be elevated up to 33-fold with increased resistance to doxorubicin as shown in (Fig. 5a, Table 2). TWIST mRNA expression was upregulated 195-fold in miRNA-212-3p MIMIC group, and up to 248-fold in FOXA1 silencer group **(**Fig. 8a), while its expression was downregulated 50-fold with increased resistance to doxorubicin as shown in (Fig. 5a, Table 2).

TP53 mRNA expression was downregulated in all examined transfected groups up to fivefold **(**Fig. 8**.b).** Noting that P53 expression was found to be elevated up to fourfold with increased resistance to doxorubicin as shown in (Fig. 5b, Table 2). BCL2 mRNA expression was downregulated in all transfected groups up to threefold (Fig. 8b). Noting that BCL2 expression was found to be elevated up to twofold with increased resistance to doxorubicin as shown in (Fig. 5b, Table 2).

The results of FOXA1 silencer and miRNA-212-3p MIMIC transfection showed complete reversal in the studied gene expression levels compared to the expression levels in the parental HepG2 cell line, suggesting that inhibition of FOXA1 help in restoring parental HepG2 cells characteristics. FOXA1 inhibition results also show that FOXA1 possesses a potential independent role in regulating epithelial-to-mesenchymal transition (EMT) genes and accordingly the metastasis process. These results suggest a possible role of miRNA-212-3p and FOXA1 in acquiring resistance to doxorubicin in HepG2 cells.

## Discussion

Liver cancer is a major global health concern, accounting for a significant proportion of cancer-related deaths. It includes various types, with hepatocellular carcinoma (HCC) being the most common, followed by intrahepatic cholangiocarcinoma and other rare subtypes. The management of advanced liver cancer poses significant challenges, with suboptimal responses to pharmacological treatments largely attributed to the multidrug resistance (MDR) phenotype [28].

In recent years, transarterial chemoembolization (TACE), a procedure that directly delivers cytotoxic chemotherapy agents to the tumor site, has provided some improvement in liver cancer treatment [6]. In addition, HAIC-FO (interventional hepatic arterial infusion chemotherapy of FOLFOX) has shown favorable survival outcomes in patients with high-risk advanced hepatocellular carcinoma, offering a potential treatment option for patients with localized high-risk factors who may benefit more from localized therapies compared to systemic treatments [29].

Despite this advancement, the development of resistance to doxorubicin, a key chemotherapeutic agent used in TACE, remains a substantial barrier to achieving effective treatment outcomes [7]. The challenges in managing hepatocellular carcinoma are also partly due to the low early detection rates, as observed in the relatively slow improvement of early-stage diagnoses for liver cancer in China compared to other cancers [30].

One of the primary mechanisms contributing to resistance is the active efflux of doxorubicin by ATP-dependent transporters, notably those from the ATP-binding cassette (ABC) transporter family, which are highly expressed in hepatocytes. These transporters play a pivotal role in transporting organic ions and xenobiotics out of cells. Their increased expression in liver cancer cell lines has been directly linked to chemotherapy resistance [25].

Moreover, alterations in microRNAs (miRNAs) have also been associated with doxorubicin resistance in liver cancer, as demonstrated by various model systems [14]. Furthermore, disruptions in epithelial-to-mesenchymal transition (EMT) genes, particularly changes in *CDH1* and *SLUG* gene expression, contribute to resistance mechanisms, not only to doxorubicin but also to other treatments, such as sorafenib [31].

Our previous studies with doxorubicin-resistant liver cancer cell lines revealed a group of transcription factors that exhibited significantly altered expression patterns compared to their parental liver cancer cell lines. Among these transcription factors, FOXA1 stood out and was selected for further analysis. The current study aimed to assess the role of modulating FOXA1 levels in doxorubicin-resistant HepG2 cells, using both a FOXA1 silencer and miRNA-212-3p mimic, to explore their potential impact on reversing drug resistance. To model the clinical setting more accurately, we developed human liver cancer cells resistant to doxorubicin (HepG2/Dox) in the laboratory, mimicking the conditions seen in TACE-treated patients. Our experiments revealed that HepG2/Dox cells were approximately 16 times more resistant to doxorubicin-induced cytotoxicity than the parental HepG2 cells, further confirming their acquired resistance.

A key feature of resistance in these cells was the significantly elevated expression of ABCB1, commonly known as the MDR1 gene, which is known to mediate drug efflux and is strongly associated with resistance to doxorubicin. Our findings demonstrated a tenfold increase in ABCB1 expression in HepG2/Dox cells compared to the control HepG2 cells, consistent with previously published studies [20, 25].

Furthermore, we observed high expression levels of FOXA1 in the resistant cells, while miRNA-212-3p, a known inhibitor of FOXA1, was downregulated by 33-fold. This inverse relationship suggests that FOXA1 plays a key role in maintaining the MDR phenotype.

FOXA1 is a well-known regulator of EMT, and its involvement in cancer progression has been established in several malignancies, including breast and liver cancers. For instance, the stable expression of FOXA1 in mesenchymal breast cancer cells was found to induce the epithelial marker E-cadherin at both mRNA and protein levels [21]. Given EMT’s role in drug resistance and apoptosis in liver cancer cells [31] we expanded our investigation to include several EMT-related genes: *CDH1*, *SLUG*, and *TWIST*, in addition to the apoptosis-related genes *TP53* and *BCL2*.

In the resistant HepG2/Dox cells, we found that the expression levels of CDH1, SLUG, P53, and FOXA1 mRNA were significantly upregulated compared to the parental HepG2 cells. Conversely, miRNA-212-3p and TWIST were downregulated. The upregulation of *CDH1 *and *SLUG* genes aligns with known genetic signatures of liver cell transformation, as documented by Stemmler et al. (2019) [31]. Interestingly, while the loss of E-cadherin is generally associated with increased metastatic potential [22], its role in liver cancer appears more complex. Studies suggest that E-cadherin expression may, in fact, facilitate the invasive potential of tumor cells within the liver environment. For example, Wei et al. (2002) reported strong E-cadherin positivity in liver metastases of gastric tumors, implying that this protein may play a role in the expansion of tumor cells within the liver. [24]

Our investigation also revealed a significant upregulation of TP53 mRNA in HepG2/Dox cells, a finding consistent with previous studies linking P53 activation to the presence of double-strand DNA breaks (DSBs) caused by doxorubicin [25] The activation of the *TP53* gene is associated with apoptosis in response to DSBs, highlighting its role in the chemotherapy response. The *BCL2* gene, known to inhibit apoptosis, was also upregulated in our resistant cells, a phenomenon commonly observed in liver cancer following treatment with other drugs like sorafenib and cabozantinib [26] The concomitant upregulation of FOXA1 and BCL2 suggests that doxorubicin may be triggering a mitochondrial apoptotic pathway in liver cancer cells.

To further evaluate the role of FOXA1 in doxorubicin resistance, we inhibited its expression in HepG2/Dox cells using both a FOXA1 silencer and miRNA-212-3p mimic. Remarkably, both interventions restored doxorubicin sensitivity to levels comparable to the parental HepG2 cells. Cytotoxicity assays and apoptosis determination using flow cytometry revealed a marked increase in doxorubicin-induced cell death following FOXA1 inhibition. Additionally, the downregulation of FOXA1 was accompanied by a significant reduction in ABCB1 expression, further supporting the hypothesis that FOXA1 may contribute to the regulation of doxorubicin efflux.

Moreover, FOXA1 inhibition reversed the expression patterns of EMT-related genes in HepG2/Dox cells. The previously upregulated E-cadherin and SLUG mRNA were downregulated, while TWIST, which had been downregulated in resistant cells, was upregulated following FOXA1 silencing. These results suggest an involvement of FOXA1 in the EMT pathway, which plays a crucial role in the development of resistance in liver cancer cells.

Similarly, apoptosis-related genes were modulated by FOXA1 inhibition. TP53 and BCL2 mRNA, both upregulated in resistant cells, were downregulated following FOXA1 silencing, indicating a possible link between FOXA1 and the apoptotic pathway. These findings were confirmed by transfecting HepG2/Dox cells with miRNA-212-3p mimic, which also led to a downregulation of FOXA1, ABCB1, E-cadherin, and SLUG mRNA, while increasing TWIST expression and reducing TP53 and BCL2 levels.

Finally, combining FOXA1 silencing or miRNA-212-3p mimic with doxorubicin resulted in a fourfold increase in apoptosis induction in HepG2/Dox cells, suggesting that targeting FOXA1 can effectively restore drug sensitivity in resistant liver cancer cells. This observation aligns with the work of Yuan et al. (2020) [15], who demonstrated the role of FOXA1 in promoting cell proliferation and suppressing apoptosis in liver cancer via the anterior gradient 2 (AGR2) and miR-212-3p pathway. Additionally, our findings resonate with the study by Kumar et al. (2021) [22], which highlighted FOXA1 as a determinant of drug resistance in hormone-dependent cancers. The parallels between FOXA1's role in breast cancer and liver cancer suggest that targeting this gene could offer a promising approach for overcoming resistance in multiple cancer types. We emphasize the need to validate our findings in other liver cancer cell lines, especially HCC-specific cell lines to confirm the role of FOXA1 in the development of resistance in different types of liver cancer.

## Conclusion

In conclusion, liver cancer remains a significant clinical challenge, with resistance to pharmacological treatments, particularly doxorubicin, posing a major obstacle. Our study provides new insights into the molecular mechanisms of doxorubicin resistance, specifically highlighting the roles of FOXA1 and miRNA-212-3p. These findings offer potential avenues for developing targeted therapies to overcome resistance and improve treatment outcomes in liver cancer patients. The modulation of FOXA1 and its downstream effectors presents a promising strategy for enhancing the efficacy of current chemotherapy regimens and offers hope for better clinical management of liver cancer.

## Data Availability

No datasets were generated or analyzed during the current study.
